# Clinical Features and Gut Microbial Alterations in Anti-leucine-rich Glioma-Inactivated 1 Encephalitis—A Pilot Study

**DOI:** 10.3389/fneur.2020.585977

**Published:** 2020-10-27

**Authors:** Xueying Ma, Lili Ma, Zhanhang Wang, Yingying Liu, Ling Long, Xiaomeng Ma, Hao Chen, Zhaoyu Chen, Xiuli Lin, Lei Si, Xiaohong Chen

**Affiliations:** ^1^Department of Neurology and Multiple Sclerosis Research Center, The Third Affiliated Hospital, Sun Yat-sen University, Guangzhou, China; ^2^Department of Neurology, Guangdong 999 Brain Hospital, Guangzhou, China

**Keywords:** anti-leucine-rich glioma-inactivated 1 encephalitis, gut microbiota, dysbiosis, 16S rRNA gene sequencing, autoimmune encephalitis

## Abstract

Anti-leucine-rich glioma-inactivated 1 (anti-LGI1) encephalitis is a rare autoimmune encephalitis (AE). We investigated the clinical features and gut microbial alterations of anti-LGI1 encephalitis. Fifteen patients newly diagnosed with anti-LGI1 encephalitis were recruited in the study prior to the administration of immunotherapy. The control group contains 25 well-matched healthy controls (HCs). All participants were Han Chinese from South China. Their clinical data and fecal samples were collected. The diversity and composition of gut microbiota were analyzed by 16S ribosomal RNA (16S rRNA) gene sequencing. The results showed that anti-LGI1 encephalitis was characterized by cognitive impairment, faciobrachial dystonic seizures, hyponatremia, and psychiatric symptoms. Abnormal EEG and brain MRI were presented in 9 and 10 patients, respectively. Compared to HCs, the anti-LGI1 encephalitis patients exhibited a decreased microbial diversity and an altered overall composition of gut microbiome. At the phylum level, anti-LGI1 encephalitis patients exhibited a higher abundance of *Proteobacteria* and a lower abundance of *Firmicutes*. The alterations in the phylum level were associated with autoimmune and inflammatory disorders. At the genus level, there was an increase in *Sphingomonas, Anaerofustis, Succinvibrio, Clostridium*, and *SMB53* (genera related to movement disorders, psychiatric diseases, and with proinflammatory effects). However, the *Faecalibacterium, Roseburia, Lachnospira, Ruminococcus*, and *Blautia* [genera with ability to produce short-chain fatty acids (SCFAs)] were obviously reduced in the patient group. Our results suggest that anti-LGI1 encephalitis is characterized by special clinical features and is accompanied by alterations in specific gut microbiota. For the limited sample size and non-applicability to other populations, further studies are warranted to explore the relationships between gut microbiota and anti-LGI1 encephalitis.

## Introduction

Autoimmune encephalitis (AE) is a group of immune-mediated inflammatory neurological diseases with antibodies against synaptic receptors, intracellular antigens, ion channels, or other neuronal cell-surface proteins (1, 2). Antibodies against leucine-rich glioma-inactivated 1 (anti-LGI1) result in a subtype of AE, and define the most frequent cause of autoimmune limbic encephalitis (ALE) (2, 3). ALE is a specific syndrome of AE that is characterized by subacute disturbances of memory, behavior and mood, and seizures. With the reported incidence of 0.83/million/year (in Netherlands in 2015), anti-LGI1 encephalitis most frequently occurs in the elderly with a male predominance. Its main clinical features include faciobrachial dystonic seizures (FBDS), serum hyponatremia and other symptoms of ALE (4, 5). Studies have established that around 90% of anti-LGI1 patients carry the human leukocyte antigen (HLA) DRB^*^07:01 and DQA1^*^02:01 (6). However, tumors are infrequent in patients. Environmental factors are essential triggers in many immune-mediated diseases, but they have been rarely investigated in this disorder. The pathogenesis of anti-LGI1 encephalitis remains obscure. In treatment, although promotion of immunotherapy takes effect in majority of patients, the spatial disorientation is hard to eliminate completely, and the relapse rate is 27–35% after a ≥2-year follow-up (3).

Gut microbiome refers to microbial organisms and their genetic material in the gastrointestinal tract of the host, among which most are bacteria. It is now widely considered as a virtual organ of the body, and *Bacteroidetes* and *Firmicutes* phyla are the two major phyla of the gut microbiota in healthy adults (7). The gut microbiota, comprising far more genes than human genomes, have been linked tightly to human health and disease (8). It has been shown that the gut microbiome can interplay with the brain through immune, endocrine, and nervous pathways. The microbiota–gut–brain axis can lead to the development of neurological disorders (9, 10). Varrin-Doyer et al. confirmed a marked sequence homology between the AQP p63-76 and p204-217 of the adenosine triphosphate-binding cassette-transporter permease expressed by *Clostridium perfringens*. It proves that the gut microbiota may be important as triggers in neurological autoimmunity (11). Therefore, there is a necessity for more studies aimed at evaluating the roles of gut microbiota in neurological autoimmune disorders including AE. Recently, Gong et al. observed an altered intestinal flora in 30 Chinese anti-NMDAR encephalitis patients compared with healthy controls. The study identified certain bacteria differentially distributed among patients in the acute phase, and those with or without relapse (12). Alterations in the composition of gut microbiota have also been reported in other autoimmune disorders such as multiple sclerosis (13) and neuromyelitis optica spectrum disorders (NMOSD) (14). However, in another study with 23 anti-N-methyl-D-aspartate receptor (anti-NMDAR) encephalitis patients from Germany, it showed that their gut microbiome were similar to that of the healthy group (15). The presence of a relationship between the gut microbiota and AE is controversial, and more studies are needed to elucidate it.

In this study, we investigated the clinical manifestations and analyzed the gut microbiota in newly diagnosed anti-LGI1 encephalitis patients. The results preliminarily identified the specificity of anti-LGI1 encephalitis in clinical features and intestinal flora. It may shed light on the relationships between gut microbiota and different AEs, and their potential for use in the diagnosis, prevention, and treatment of anti-LGI1 encephalitis.

## Materials and Methods

### Study Participants and Sample Collection

Signed informed consent was obtained from all study participants at enrollment. Ethical approval was obtained from the Medical Ethics Committee of the Third Affiliated Hospital of Sun Yat-sen University (approval ID: [2018]02-363-01).

**Figure 1:**
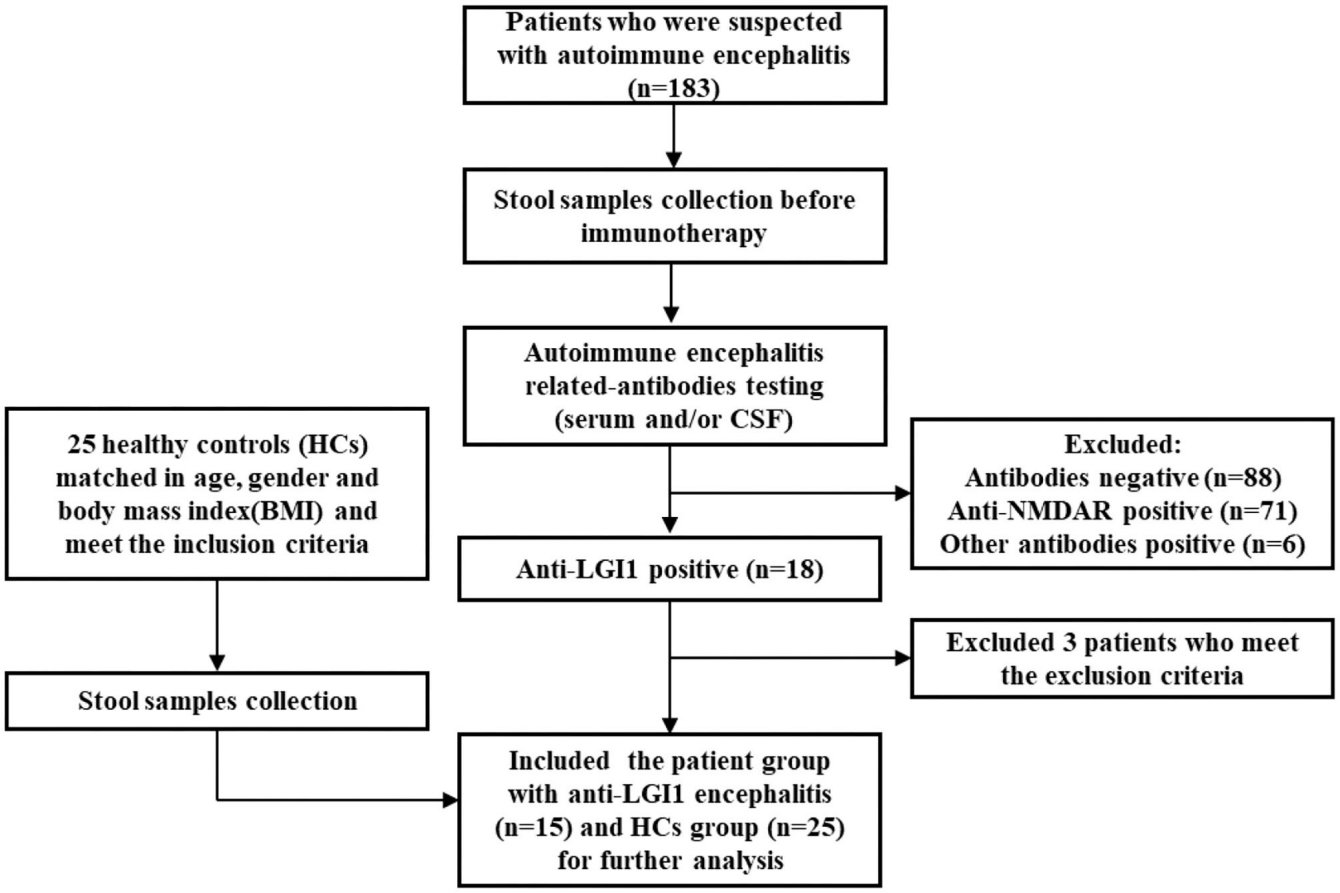
Flowchart illustrating the enrollment of anti-leucine-rich glioma-inactivated 1 (anti-LGI1) encephalitis patient group and healthy controls (HCs).

Enrollment details are shown in Figure 1. From August 2018 to October 2019, a total of 183 patients with suspected autoimmune encephalitis were admitted to the Department of Neurology of the Third Affiliated Hospital of Sun Yat-sen University. Their stool samples were collected on the morning after admission and before immunotherapy. The inclusion criteria were: (i) age 18 or older; (ii) subacute onset of memory disturbance, changed mental status, seizures, or psychiatric symptoms. Antibody testing was then conducted. Eighteen patients were positive for LGI1 antibodies (16). Among them, three patients were excluded according to the exclusion criteria: (i) who took antibiotics, probiotics, or prebiotics within 1 month before sampling; (ii) with previous autoimmunological diseases, psychiatric disorders, neurodegenerative disorders, gastrointestinal diseases, or bowel surgery. Fifteen patients newly diagnosed with anti-LGI1 encephalitis were, therefore, enrolled as the patient group for further study. The healthy controls (HCs) group was made up of 25 healthy people from the Medical Examination Center of the Third Affiliated Hospital of Sun Yat-sen University Hospital. They were matched with the patient group in age, gender, and body mass index (BMI), and had to fulfill the following criteria: with normal ranges in routine laboratory tests such as fasting glucose, blood urine, blood lipids, liver and kidney functions, and stool.

After collection, the stool samples were stored at −80°C immediately for the following analysis.

### Clinical Data Collection

The demographic information, clinical manifestations, neuropsychological assessments, laboratory findings, electroencephalography (EEG) examination, and brain MRI results were collected. The abnormal MRI in this study was defined by abnormal signaling in medial temporal lobe on fluid attenuated inversion recovery (FLAIR) or T2 sequences. The Minimum Mental State Examination (MMSE) (score range: 0–30) and Montreal Cognitive Assessment Scale (MoCA) (score range: 0–30) were used to assess the cognitive status of the patients by experienced neurologists. The patient group received a series of regular laboratory tests with a focus on the result of serum sodium. In addition, the autoimmune encephalitis-related antibodies [blood and/or cerebrospinal fluid (CSF), including NMDAR, LGI1, and other neuronal surface- or synaptic protein-related antibodies] were undertaken by an indirect immunofluorescence assay with standard kits (EUROIMMUN Medizinische Labordiagnostika, Lübeck, Germany). Classical onconeural antibodies (CV2, Hu, Yo, Ri, Ma, amphiphysin, and so on) were also tested.

### DNA Extraction, 16S rRNA PCR, and Sequencing

The protocol of 16S rRNA sequencing was based on our previous studies (17). The QIAamp DNA Stool Mini Kit (Qiagen, Germany) was applied to extract the bacterial DNA from stool samples. The V4 region of 16S rRNA was amplified by polymerase chain reaction (PCR) under the following reaction conditions: an incubation at 98°C for 3 min; followed by 30 cycles of denaturation at 98°C for 45 s, annealing at 55°C for 45 s, and extension at 72°C for 45 s; followed by a final elongation step at 72°C for 7 min. Amplicons were purified by AmpureXP beads (AGENCOURT). For quality control, the purified amplicons were quantified by the Agilent 2100 bioanalyzer instrument (Agilent DNA 1000 Reagents, to determine the average molecule length) and real-time quantitative PCR (EvaGreen TM, to quantify the library). The paired-end sequence of qualified libraries was then performed on a MiSeq system, with the sequencing strategy PE250 (PE251 + 8 + 8 + 251) or PE300 (PE301 + 8 + 8 + 301) (MiSeq Reagent Kit).

### Statistical Analysis

Clinical data analysis was performed using the Graphpad Prism 8.0 software with unpaired two-tailed Student's *t*-test or chi-square test. Continuous variables were expressed as the median (range) or mean ± SD. Sequencing data was analyzed with the R package (version 2.15.3). The Quantitative Insights into the Microbial Ecology (QIIME) pipeline was performed to analyze the alpha and beta diversity (18). Alpha diversity was estimated by the Chao 1, Simpson, Shannon, ACE, and Observed Species. Differences between the two groups were analyzed with Student's *t*-test. The beta diversity was evaluated by principal coordinate analysis (PCoA) and Venn diagram. PCoA was evaluated by the Bray–Curtis distances to graphically identify different microbial community structures. The linear discriminant analysis (LDA) effect size (LEfSe) analysis was applied to identify differentially abundant taxa between patients and the HCs, by using the non-parametric factorial Kruskal–Wallis sum-rank test and then the (unpaired) Wilcoxon rank-sum test (19). The LDA threshold was >4. Statistical significance was considered at *p* < 0.05.

## Results

### Clinical Features of Anti-LGI1 Encephalitis

**Figure 2:**
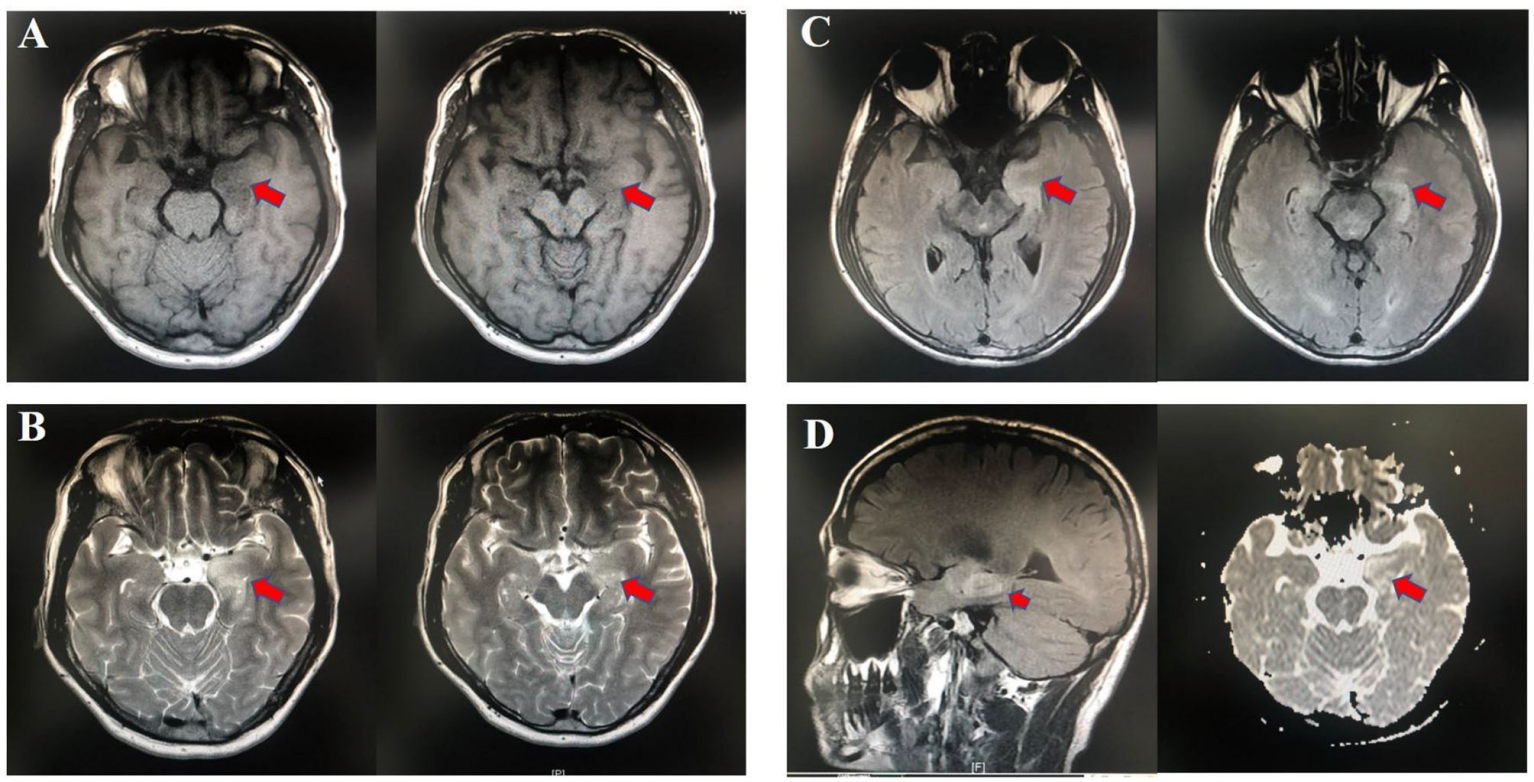
Abnormal MRI signaling (Red arrows) in the left hippocampus and parahippocampal gyrus of one patient with anti-LGI1 encephalitis. **(A)** TI-weighted MRI. **(B)** T2-weighted MRI. **(C)** Aixal FLAIR sequences. **(D)** Sagittal FLAIR MRI and axial DWI. MRI, magnetic resonance imaging; FLAIR, fluid attenuated inversion recovery; DWI, diffusion-weighted imaging.

Fifteen patients newly diagnosed with anti-LGI1 encephalitis before immunotherapy and 25 age-, gender-, and BMI-matched HCs were recruited. Majority (14 of 15) of patients were at an acute phase of the disease when stool samples were collected. All the patients presented with cognitive disorders, while 10 (67%) presented with FDBS. Nine of the 15 patients (60%) had hyponatremia, and the mean serum sodium concentration of all patients was 132.90 mmol/L. The MMSE and MoCA scores were 22.07 ± 4.04 and 16.33 ± 5.86, respectively. Thirteen patients underwent EEG, among whom nine exhibited abnormalities, and slow basic rhythm was common. Ten patients manifested with an abnormal brain MRI. The most common abnormalities were hyperintense on T2 and FLAIR imaging of unilateral or bilateral medial temporal lobe and hippocampus. The MRI features of an anti-LGI1 encephalitis patient in our cohort were presented (Figure 2). The demographics and clinical data of patients and HCs were collected in detail and illustrated in Table 1.

**Table 1:** Demographic and clinical characteristics of participants.

	**Anti-leucine-rich glioma-inactivated 1 (anti-LGI1) encephalitis**	**Healthy controls**	***p-*value**
Sample size	15	25	
Nationality (Han)	15/15 (100%)	25/25 (100%)	
Age (year, mean ± SD)	54.33 ± 12.26	55.32 ± 11.19	0.796^a^
Sex (male/female)	9/6	16/9	0.800^b^
BMI (kg/m^2^, mean ± SD)	22.12 ± 2.03	23.01 ± 2.23	0.215^a^
Onset to visit [days, median (range)]	30 (7–120)		
Symptoms			
FBDS	10/15 (67%)		
Memory deficit	15/15 (100%)		
Spatial disorientation	9/15 (60%)		
Psychiatric symptoms	11/15 (73%)		
Autonomic disorders	5/15 (33%)		
Sleep disturbance	6/15 (40%)		
Hyponatremia	9/15 (60%)		
Tumors	0/15 (0%)		
MMSE score (mean ± SD)	22.07 ± 4.04		
MoCA score (mean ± SD)	16.33 ± 5.86		
Serum			
LGI1 antibody	15/15 (100%)		
Paraneoplastic antibodies	0/15 (0%)		
Na^c^ (mmol/L, mean ± SD)	132.90 ± 6.56		
CSF
LGI1 antibody	8/13 (62%)		
Abnormal EEG	9/13 (69%)		
Abnormal brain MRI	10/15 (67%)		

a *Student's t-test*.

b *Chi-square test*.

c *Serum Na normal range: 135–145 mmol/L*.

### Patients With Anti-LGI1 Encephalitis Showed Reduced Microbial Diversity

**Figure 3:**
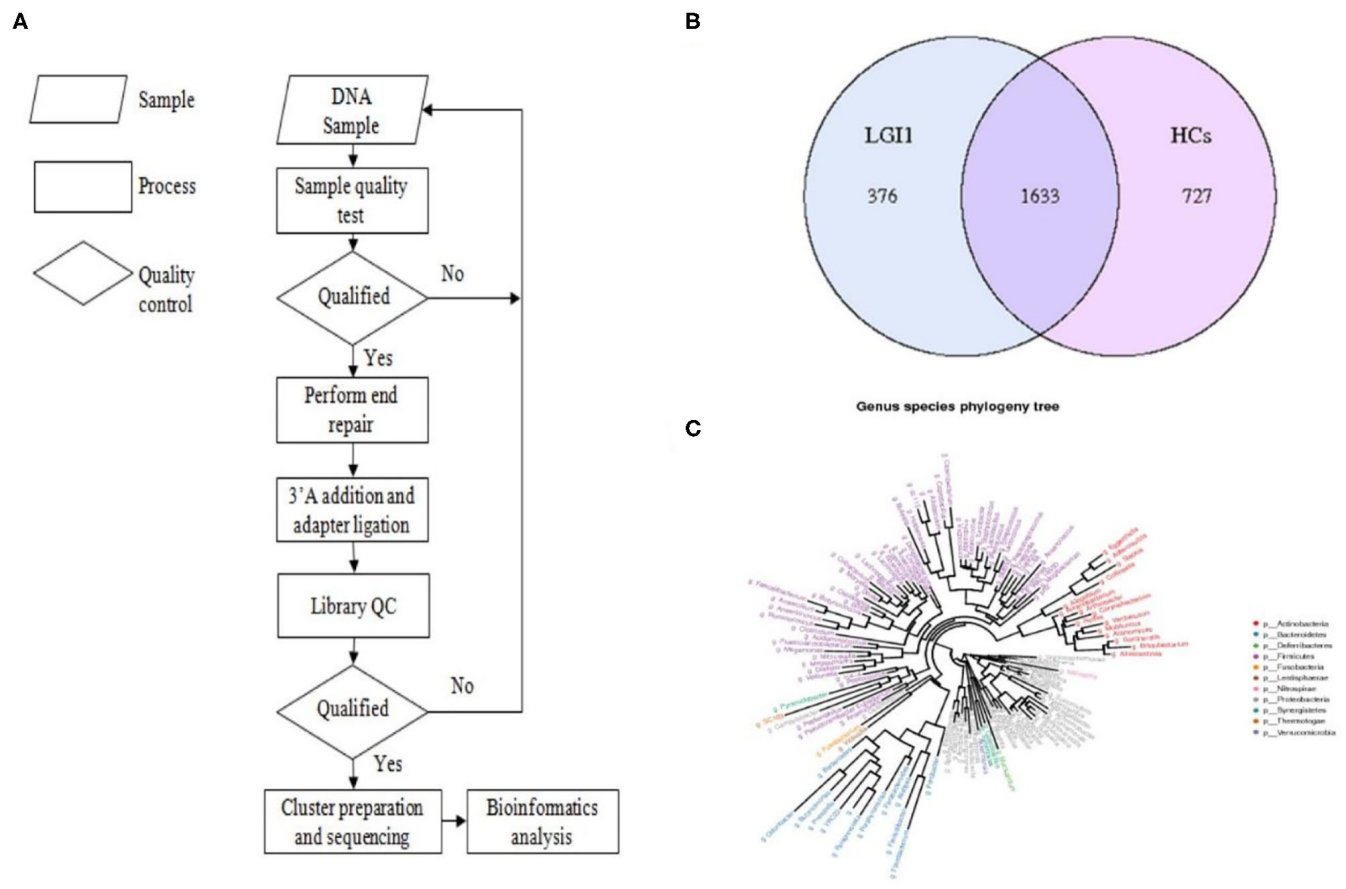
Gut microbial composition differences in anti-LGI1 encephalitis and HCs. **(A)** Flowchart illustrating the methods. **(B)** Venn diagram representation of the gut microbiota between anti-LGI1 encephalitis patients and HCs. Operational taxonomical unit. OTUs, 376 and 727, were found to be unique to the anti-LGI1 encephalitis patients and HCs, respectively. The shared OTUs were 1,633, and accounted for 81.3% (1633/2009) and 69.2% (1,633/2,360) in the patient and HCs samples, respectively. **(C)** Genus-species phylogeny tree of all subjects.

**Figure 4:**
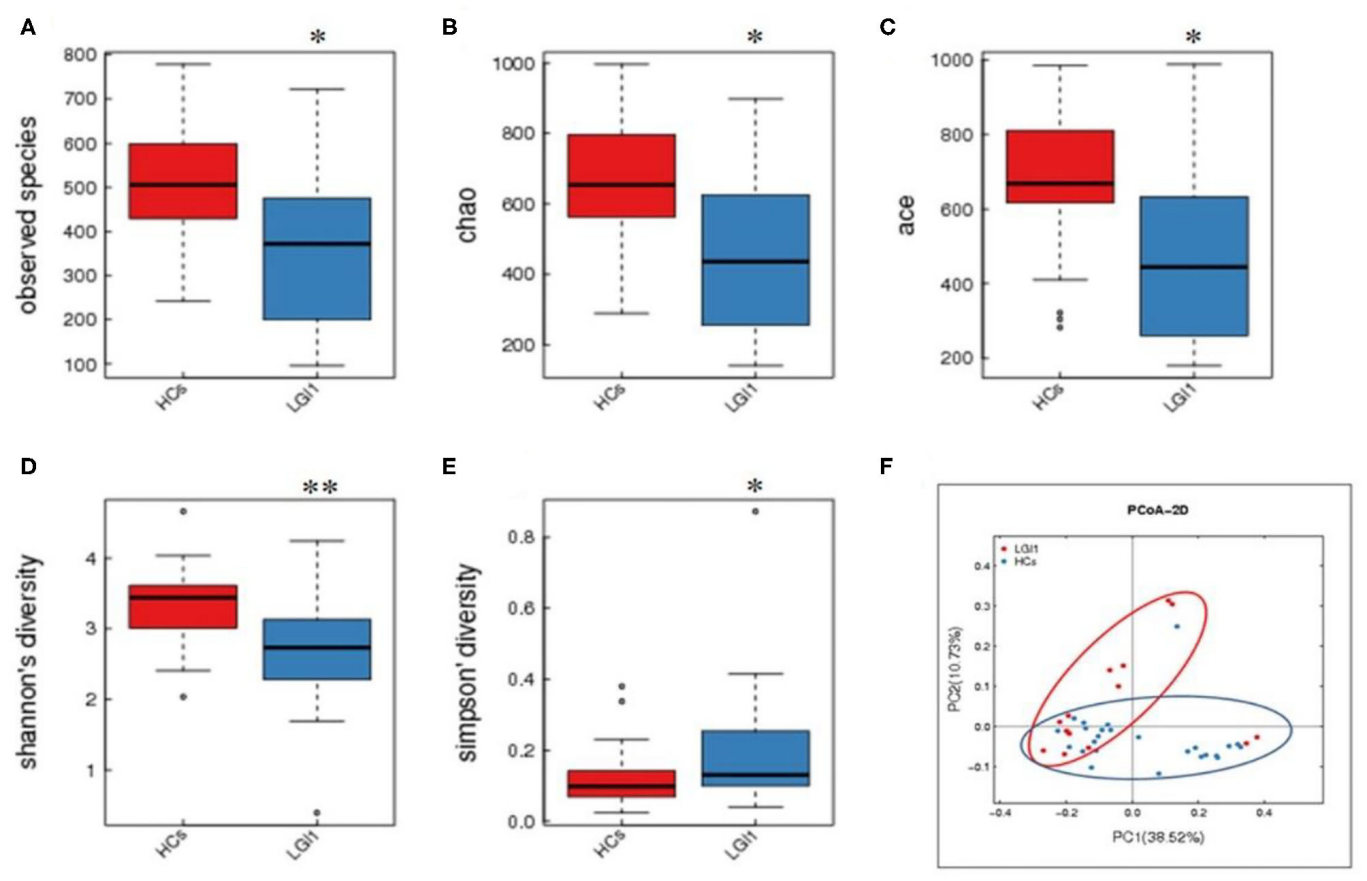
Reduced microbial diversity and altered overall microbial composition detected in anti-LGI1 encephalitis. **(A–E)** Alpha-diversity in anti-LGI1 encephalitis patients and HCs. Box plots depict reduced microbial diversity in anti-LGI1 patients according to observed species, Chao1, ace, Shannon diversity, and Simpson diversity index. Lines inside the box represent the median values, the upper and lower ranges of the box represent the interquartile range values, and the horizontal lines depict minimum and maximum values (*p < 0.05, **p < 0.01). **(F)** Beta-diversity analysis in the two groups. Weighted PCoA based on the UniFrac distance illustrate the microbial community variation between the anti-LGI1 encephalitis and HCs (pseudo-F: 3.84, *p* < 0.001).

Fecal samples from all subjects were analyzed by 16S rRNA gene amplicons sequencing (Figure 3A). The results yielded 1,646,690 high-quality reads with an average length of 253 bp. After clustering these sequencing at 97% similarity threshold, a total of 2,736 operational taxonomic units (OTUs) were obtained for the downstream study. The Venn figure depicted that 376 and 727 OTUs were unique to the anti-LGI1 encephalitis patients and HCs, respectively. Shared OTUs accounted for 81.3% (1,633/2,009) and 69.2% (1,633/2,360) in the patient and HC samples, respectively (Figure 3B). The Figure 3C showed the genus-species phylogeny tree of all subjects. In the alpha-diversity analysis, the Observed Species, Chao1, and ACE indices indicated the community richness, while the Shannon and Simpson indices indicated the community diversity. As shown in Figures 4A–E, the Simpson index was higher, while the other indices were lower in patients with anti-LGI1 encephalitis compared to the HCs group. These differences were significant. The results showed that within-sample microbial diversity reduced in the anti-LGI1 encephalitis patients.

### Altered Overall Microbial Composition in Anti-LGI1 Encephalitis Patients

To further assess the overall differences of the microbial composition in anti-LGI1 encephalitis patients, the beta-diversity analysis was performed. In the PCoA based on weighted UniFrac distance, a significant difference in the overall composition of microbial community was observed between the patients with anti-LGI1 encephalitis and HCs (pseudo-F: *n, p* < 0.001, Figure 4F).

### Gut Microbiota Differentially Abundant in Anti-LGI1 Encephalitis vs. HCs

**Figure 5:**
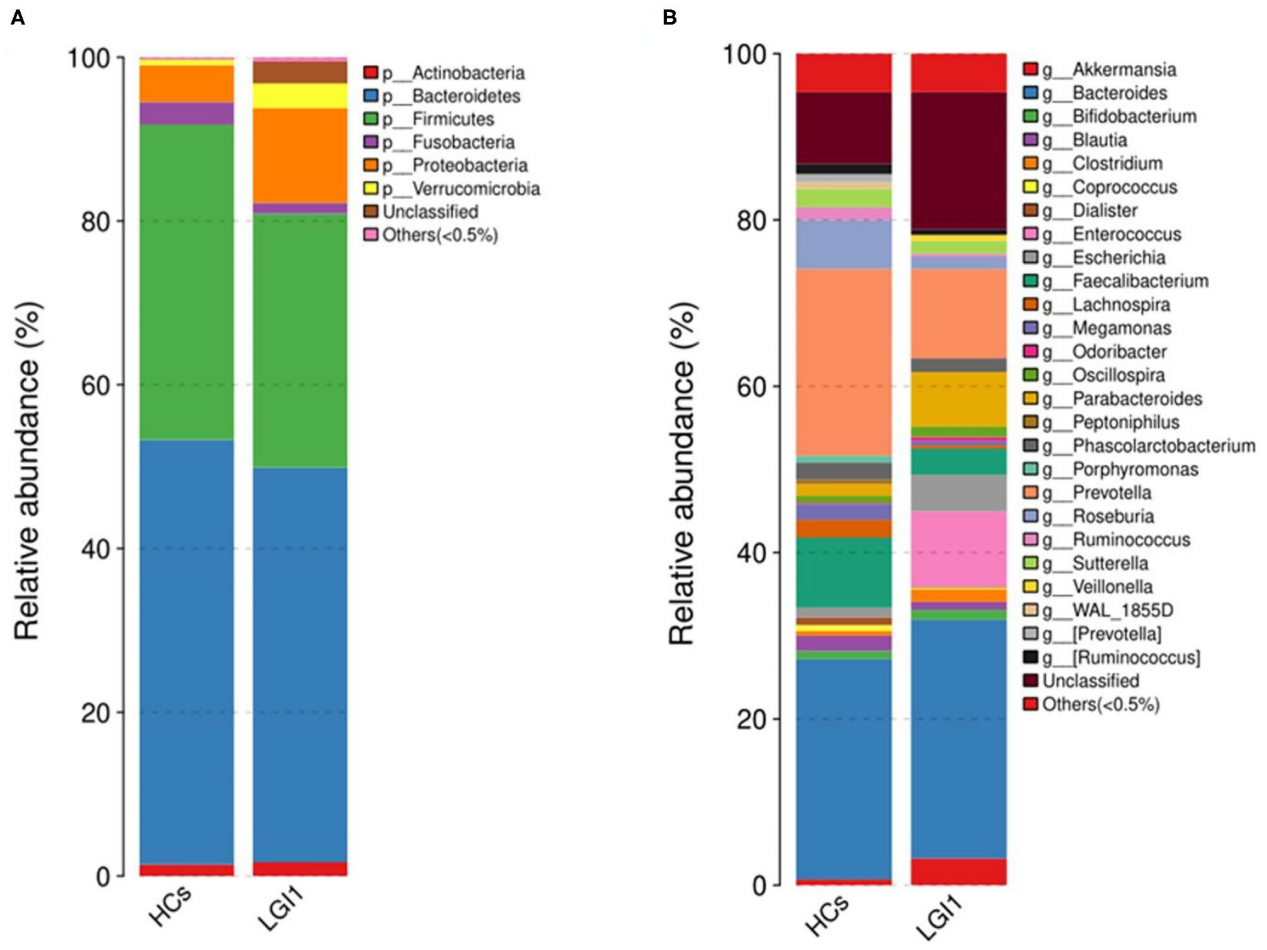
Relative abundances of microbial communities in anti-LGI1 encephalitis patients and HCs at **(A)** phylum level and **(B)** genus level.

Apart from the differences in the overall microbial composition, the gaps in microbial abundance between the two groups were also detected at all levels. At the phylum level, *Bacteroidetes* and *Firmicutes* constituted the two most dominant entities in gut microbiota (Figure 5A), while at the genus level, *Bacteroides, Prevotella, Akkermansia, Faecalibacterium, Roseburia*, and *Parabacteroides* were the most abundant in the two groups (Figure 5B). In addition, the anti-LGI1 encephalitis patients had a higher abundance of the *Proteobacteria* phylum, and a lower abundance of the *Bacteroidetes* and *Firmicutes* phylum, when compared with HCs.

**Figure 6:**
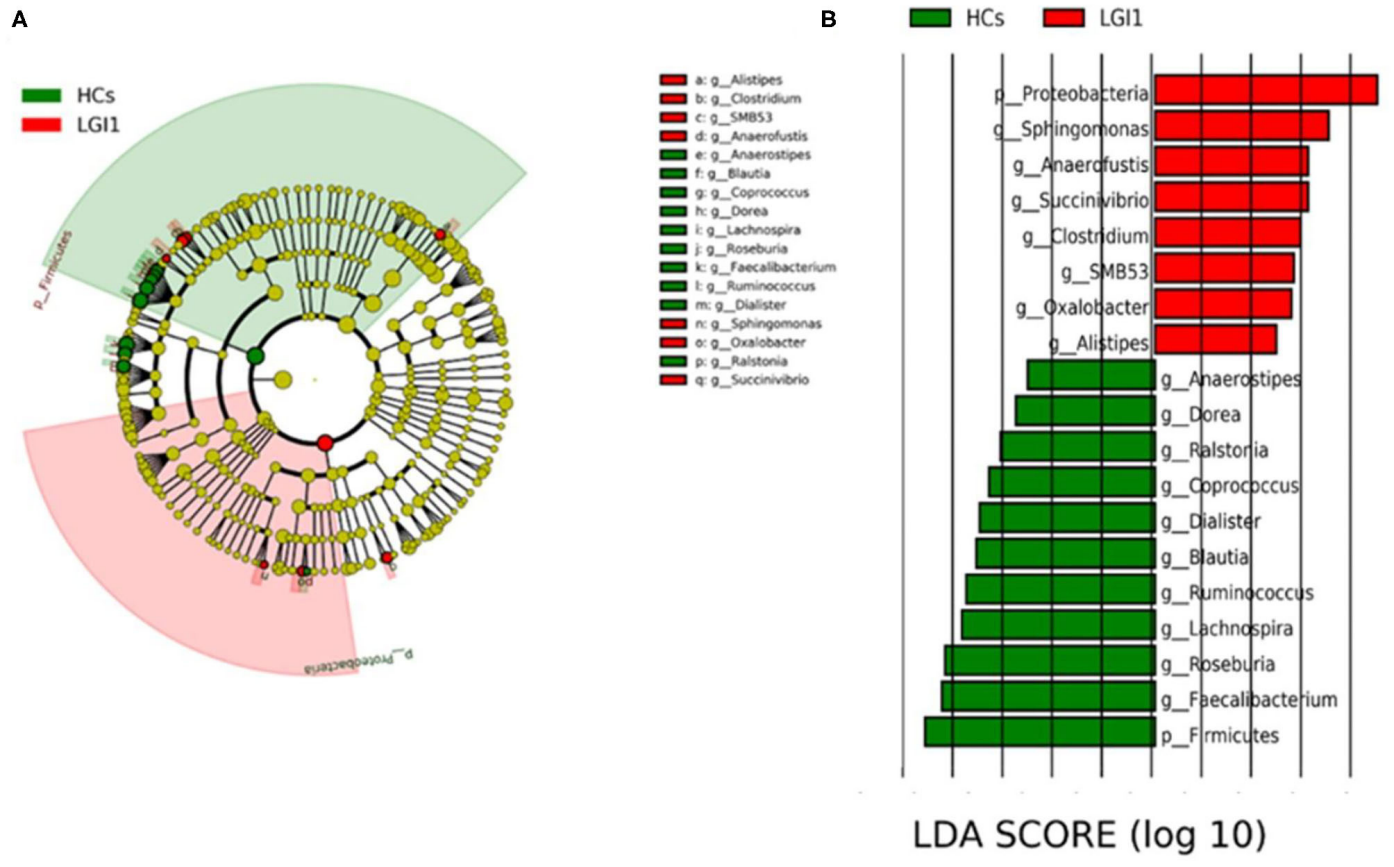
Effect size (LEfSe) analysis identified the taxonomic composition differences in patients with anti-LGI1 encephalitis compared with HCs. **(A)** A cladogram showing the phylogenetic distribution of gut microbiota related to the anti-LGI1 encephalitis (red) and HCs (green). **(B)** Linear discriminant analysis (LDA) scores indicating the significant bacterial differences between the anti-LGI encephalitis (red) and healthy groups (green). The LDA scores (log10)> 2 and *p* < 0.05 are listed.

To further seek for the significant increased bacteria in patients with anti-LGI1 encephalitis or HCs, the LEfSe was applied to conduct supervised comparisons on differentially distributed taxa. As presented in Figure 6, the relative abundance of phylum *Proteobacteria* was significantly higher in the patient group compared to the healthy group, while the phylum *Firmicutes* was notably enriched in the HCs (*p* < 0.05, LDA score >2). The specific bacterial genera differentially distributed between anti-LGI1 encephalitis and HCs were also identified. *Sphingomonas, Anaerofustis, Succinivibrio, Clostridium*, and *SMB53* genera were remarkly outnumbered in the patients with anti-LGI1 encephalitis. *Faecalibacterium, Roseburia, Lachnospira, Ruminococcus*, and *Blautia* that overpresented in HCs were significantly deficient in the patients (*p* < 0.05, LDA score >2). These results suggest that the microbiota mentioned above at the phylum and genus levels were responsible for discriminating the anti-LGI 1 encephalitis from HCs.

## Discussion

AE is an increasingly prevalent inflammatory disorder of the central nervous system (CNS). Although complex genetic and environmental triggers have been considered involved in the disorder, its pathogenesis has not been fully elucidated (16). Intestinal microbiota has been demonstrated to bear profound influences on brain development, functions, and behaviors through the microbiota–gut–brain axis. Accumulating evidences have also indicated its effects in the CNS autoimmunity (20). Anti-LGI1 encephalitis is a type of AE whose gut microbiota remains unclear. In this study, we determined the clinical manifestations of patients newly diagnosed with anti-LGI1 encephalitis, and, for the first time, analyzed their gut microbiota.

Patients with anti-LGI1 encephalitis exhibited subacute cognitive impairment, FBDS, hyponatremia, and psychiatric symptoms as its core clinical characteristics, while abnormal EEG or brain MRI were detected in some. FBDS is a distinctive manifestation of anti-LGI1 encephalitis and was presented in 67% of the patients in this study. Whether it is one kind of seizure or a paroxysmal movement disorder is unknown (21, 22). These results are in accordance with previous reports (23, 24).

The within-sample diversity of fecal microbiota was lower in anti-LGI1 encephalitis patients compared with HCs. The species richness of gut microbiota has been proposed to construct a healthy microbial community. A loss of diversity represents a frequent characteristic of diseases-associated dysbiosis (25). The correction of this index may be therapeutic methods or a good prognostic factor in anti-LGI1 encephalitis. However, the gut microbiota in anti-NMDAR encephalitis exhibited contrast results, characterized by higher or similar alpha diversity compared with HCs (12, 15). Although both are AE subtypes, the triggers for anti-LGI1 encephalitis and anti-NMDAR encephalitis are likely to be distinct. The occurrence of Anti-NMDAR encephalitis has been associated with virus infection and the presence of tumors, especially ovarian teratomas, while anti-LGI1 encephalitis has been associated with significant genetic predisposition (2). The existence of gut dysbiosis and types of dysbiosis may be different between the two AEs. A bloom of pathogens or a lack of beneficial microbiota without a low diversity may be the main form of gut dysbiosis in anti-NMDAR encephalitis. This finding has been reported in multiple sclerosis and NMOSD (26, 27). The heterogeneity of the gut microbial indices in AEs may link to differences in the distribution of populations, clinical features, or their specific pathogenesis. However, their cause and consequences are unclear. Of note, a recent study has warned that the microbial diversity and richness may not be good indicators of gut dysbiosis. It is because they can be influenced by many confounders, some of which are difficult to control and are related with some clinical features of these diseases (28). Further studies in the effects of changed microbiota on the host are warranted.

The overall composition of microbial composition in the patient group was distinct from that in HCs, which again suggests an abnormal gut status in anti-LGI1 encephalitis. In accordance with reported results from healthy human guts (29), fecal microbiota from anti-LGI1 encephalitis patients and HCs in this study predominantly belong to the *Bacteroidetes* and *Firmicutes* phyla. Supervised comparisons with LEfSE showed that the anti-LGI1 encephalitis patients were characterized by an increase in the *Proteobacteria* phylum, while HCs had enriched *Firmicutes*. That decrease of *Firmicutes* in the patient groups has been documented in several diseases (26, 30). Additionally, gut dysbiosis defined in many autoimmune and inflammatory disorders presented with a deviation in the ratio of *Firmicutes/Bacteroidetes* (31). However, diseases-associated dysbiosis also includes the outnumbered *Proteobacteria*. Compared with *Firmicutes* and *Bacteroidetes, Proteobacteria* accounts for a smaller proportion of healthy gut flora and is far more unstable. Experimental and clinical evidences presume a positive feedback loop that the *Proteobacteria* may be a sensitive responder to acute or chronic inflammation. It leads to the expansion of *Proteobacteria*, which can exaggerate the inflammation (32). The bloom of *Proteobacteria* has been detected in studies of inflammatory bowel disease (IBD) and other autoimmune diseases such as NMOSD (26, 33). Elevation of this phylum in our results may suggest its proinflammatory effects in anti-LGI1 encephalitis. Restoring this unstable gut microbiota may cut off the feedback loop, with potential in the treatment of this disease.

At the genus level, the patient group was characterized by a significantly increased *Sphingomonas, Anaerofustis, Succinivibrio, Clostridium*, and *SMB53*, while *Faecalibacterium, Roseburia, Lachnospira, Ruminococcus*, and *Blautia* were obviously decreased. In a previous report, feces from three subgroups of anti-NMDAR encephlitis patients showed enrichment of *Bacteroides, Streptococcus* and *Parabacteroids*, and *Fusobacterium* genera, respectively (12). The differences in specific microbial lanscapes between the two AEs indicate that the gut microbiota may be an effective marker for the differential diagnosis of anti-NMDAR encephalitis and anti-LGI1 encephalitis.

*Sphingomonas* within the phylum *Proteobacteria*, is a Gram-negative aerobe with low pathogenicity and can be found in many environments (34). They have been found increased in patients with Parkinson's disease (35). *Anaerofustis* is a Gram-positive and strictly anaerobic bacterial genus. Its alteration has not been mentioned in the human gut with diseases before (36). It is possible that the outgrowth of this pathogen is exclusive for anti-LGI1 encephalitis and serves as a potential biomaker for this disorder. *Succinivibrio* genus within the *Proteobacteria* phylum has been reported increased in patients with schizophrenia (37). Among the species within the *Clostridium* genera, *Clostridium perfringens* has been postulated to have possible molecular mimic and proinflammatory effects in the pathogenesis of NMOSD, and it triggers multiple sclerosis by producing toxin (38, 39). However, *Clostridium* has also been demonstrated to induce regulatory T cells (Treg cells). A deficency in specific species have been implicated in autoimmune disorders such as multiple slcerosis (27). It suggests that functions of the *Clostridium* genus vary at the species level. Further studies on members within this genus in anti-LGI1 encephalitis are needed. Recent studies have reported that some bacteria are associated with not only the onset of diseases but also with the certain clinical symptoms (40–42). Given the fact that the *Sphingomonas* genera increased in both anti-LGI1 encephalitis and Parkinson's disease, we made a bold assumption that the *Sphingomonas* may link to the extrapyramidal symptoms or even the pathomechanisms of FBDS. Besides, the increase in *Succinivibrio* in both anti-LGI1 encephalitis and schizophrenia may be associated with psychiatric symptoms. However, the relationships between gut microbiota and certain clinical manifestation are just bold guesses that we made. Overload in these certain genera may contribute to the development of anti-LGI1 encephalitis or even link to its specific symptoms. However, the mechanisms by which these genera act as triggers, promoters, or responsers in the pathogenesis of anti-LGI1 encephalitis are unclear. Further studies are expected to evaluate the gut microbiota among patient subgroups with different clincial manifestations with a large cohort.

Through fermentation, the *Faecalibacterium, Roseburia, Lachnospira, Ruminococcus*, and *Blautia* genera within the *Firmicutes* phylum can convert the dietary fibers to the short-chain fatty acids (SCFAs) such as acetate, butyrate, and propionate. Therefore, they are members of the SCFAs-producers (43). The gut microbiota play important roles in brain and behavior development through different mechanisms. They produce microbial metabolites that are important for host health. SCFAs cannot only act as energy scources but also recruit leukocytes to inflammation area, promote T cell differentiation toward the Treg cells, modulate microglia maturation, influence the permeability of the blood–brain barrier, and so on (43, 44). Processes of CNS autoimmune diseases are considered to involve the immune activation, the antibody production and the blood–brain barrier disruption, which allows IgG to transverse it (45). Neurological autoimmune diseases such as NMOSD and multiple sclerosis show deplepted SCFAs-producers that related to disease severity (26, 46). The depletion of these genera may participate in the pathogenesis of anti-LGI1 encephalitis, and microbial metabolites especially SCFAs should be further studied in the disorder.

As the first detection of gut microbiome in patients with anti-LGI1 encephalitis, this study shows a specific microbiota landscape in the patient group that is distinct from HCs. The bacteria that were significantly altered in the patient group may be involved in the pathogenesis of anti-LG1 encephalitis. However, there are several limitations in this study. First, since it is a preliminary study, the sample size was limited. Further studies with an expanded samples size will be done to evaluate the relationships between gut microbiota, and different clinical characteristics or different disease phases of anti-LGI1 encephalitis. Second, all the study participants were Han Chinese from South China. Due to the diversity of dietary habits and genetic diversities, our conclusion may be specific to this population and cannot be simply applied for other populations. Studies from different regions are needed to explore specific bacteria of the disorder. Third, although the samples were collected before immunotherapy, the intestinal microbiota could have been influenced by other confounders such as diet, exercise, anti-epileptic drugs, and antipsychotic medication. Fourth, we did not detect the fecal level of SCFAs or other microbial metabolites. Detection of these products may help us to understand the associations of the microbiota–gut–brain axis and anti-LGI1 encephalitis comprehensively. Further metabolomic analyses are needed. Besides, we did not clarify whether there is a cause–effect relationship between the gut microbiota and anti-LGI1 encephalitis. Related experiments involving animal models will be of utmost value in deciphering this disorder.

## Conclusion

Our findings illustrate specific clinical features of anti-LGI1 encephalitis and provided preliminary evidences of gut dysbiosis. This may help distinguish the disorder from other AEs. Moreover, gut microbiota are expected to be potential therapeutic targets of anti-LGI1 encephalitis.

## Data Availability Statement

The original contributions presented in the study are publicly available. This data can be found in the National Center for Biotechnology Information (NCBI) BioProject database with the project number PRJNA663653.

## Ethics Statement

The studies involving human participants were reviewed and approved by the Medical Ethics Committee of the Third Affiliated Hospital of Sun Yat-sen University (approval ID: [2018]02-363-01). The patients/participants provided their written informed consent to participate in this study.

## Author Contributions

XC, HC, XuM, and ZW designed the experiments. XuM, LL, and YL performed the metagenomic analysis and analyzed the data. XuM, ZW, ZC, and XiM collected the fecal samples. XuM and HC drafted the manuscript. XC, XiM, XL, LM, and LS revised the manuscript. All authors have participated in the review and editing.

## Conflict of Interest

The authors declare that the research was conducted in the absence of any commercial or financial relationships that could be construed as a potential conflict of interest.
